# Developmental fluoride neurotoxicity: an updated review

**DOI:** 10.1186/s12940-019-0551-x

**Published:** 2019-12-19

**Authors:** Philippe Grandjean

**Affiliations:** 1 Department of Environmental Health, Harvard T.H. Chan School of Public Health, Boston, MA 02115 USA; 2 Department of Public Health, University of Southern Denmark, Odense, Denmark

**Keywords:** Cognitive disorder, Dental caries, Drinking water, Fluoridation, Fluoride poisoning, Intellectual disability, Neurotoxic disorder, Prenatal exposure delayed effects

## Abstract

**Background:**

After the discovery of fluoride as a caries-preventing agent in the mid-twentieth century, fluoridation of community water has become a widespread intervention, sometimes hailed as a mainstay of modern public health. However, this practice results in elevated fluoride intake and has become controversial for two reasons. First, topical fluoride application in the oral cavity appears to be a more direct and appropriate means of preventing caries. Second, systemic fluoride uptake is suspected of causing adverse effects, in particular neurotoxicity during early development. The latter is supported by experimental neurotoxicity findings and toxicokinetic evidence of fluoride passing into the brain.

**Method:**

An integrated literature review was conducted on fluoride exposure and intellectual disability, with a main focus on studies on children published subsequent to a meta-analysis from 2012.

**Results:**

Fourteen recent cross-sectional studies from endemic areas with naturally high fluoride concentrations in groundwater supported the previous findings of cognitive deficits in children with elevated fluoride exposures. Three recent prospective studies from Mexico and Canada with individual exposure data showed that early-life exposures were negatively associated with children’s performance on cognitive tests. Neurotoxicity appeared to be dose-dependent, and tentative benchmark dose calculations suggest that safe exposures are likely to be below currently accepted or recommended fluoride concentrations in drinking water.

**Conclusion:**

The recent epidemiological results support the notion that elevated fluoride intake during early development can result in IQ deficits that may be considerable. Recognition of neurotoxic risks is necessary when determining the safety of fluoride-contaminated drinking water and fluoride uses for preventive dentistry purposes.

## Background

In 2006, the U.S. National Research Council (NRC) evaluated the fluoride standards of the Environmental Protection Agency (EPA) and concluded that fluoride can adversely affect the brain through both direct and indirect means, that elevated fluoride concentrations in drinking-water may be of concern for neurotoxic effects, and that additional research was warranted [1]. At the time, and continuing through today, the EPA’s Maximum Contaminant Level Goal (MCLG) for fluoride was 4.0 mg/L that aimed at protecting against crippling skeletal fluorosis, which is still considered to be the critical adverse health effect from fluoride exposure [2]. Following the NRC review, evidence has accumulated that the developing human brain is inherently much more susceptible to injury from neurotoxic agents, such as fluoride, than is the adult brain [3]. A review and meta-analysis published in 2012 [4] assessed a total of 27 research reports, all but two of them from China, on elevated fluoride exposure and its association with cognitive deficits in children. All but one study suggested that a higher fluoride content of residential drinking water was associated with poorer IQ performance at school age. Only a couple of these studies had been considered by regulatory agencies [1, 5]. As much additional evidence has emerged since then, it seems appropriate to update the assessment of potential human neurotoxicity associated with elevated fluoride exposure, especially during early development.

The present review first outlines the importance of drinking water as a source of fluoride exposure, followed by the toxicokinetics of fluoride absorbed into the body, including passage through the placenta and the blood-brain barrier, and finally a brief summary of the experimental evidence of developmental neurotoxicity. All of this evidence supports the plausibility that elevated fluoride exposure in early life may cause adverse effects on the brain. The main part of this review addresses the epidemiological studies of fluoride neurotoxicity, with a focus on children and the dose-dependent impact of prenatal and early postnatal exposures.

### Potential sources of fluoride exposure

Fluoride occurs in many minerals and in soil [6], thus also in groundwater; the average concentration in the U.S. is 0.26 mg/L [7]. Since the mid-1940s, fluoride has been added to many community water supplies with the aim of preventing tooth decay [8]. In the U.S., fluoridation is recommended at a concentration of 0.7 mg/L [9]. Water fluoridation is applied in several other countries as well, such as Australia, Brazil, Canada, Chile, Ireland, New Zealand, and the United Kingdom. For adults in the U.S., fluoridated water and beverages contribute an average of about 80% of the daily total fluoride intake (estimated to average 2.91 mg) in fluoridated communities [10]. In a Canadian study of pregnant women [11], water fluoridation was the major predictor of urinary fluoride excretion levels, with creatinine-adjusted concentrations of 0.87 mg/L and 0.46 mg/L in fluoridated (0.6 mg/L water) and non-fluoridated (0.12 mg/L) communities.

In addition to fluoridated water and other forms of caries prevention, tea is an important source of fluoride exposure, even if prepared with deionized water [12, 13]. Additional sources of fluoride intake include certain foods (such as sardines), industrial emissions, supplements, pesticide residues, and certain pharmaceuticals that can release fluoride [1]. Few studies provide population-based data on fluoride exposure, although national data on plasma-fluoride concentrations are available from a recent NHANES study in the U.S. [14].

### Uptake, distribution and retention

Approximately 75–90% of ingested fluoride is absorbed and readily distributed throughout the body, with approximately 99% of retained fluoride being bound in calcium-rich tissues such as bone and teeth [6, 15] as well as the calcified parts of the pineal gland [16]. Fluoride also crosses the placenta and reaches the fetus [1, 6] and the amnionic fluid [17]. The fluoride concentration in breast milk is low, generally less than 0.01 mg/L [1, 18], and formula can therefore contribute much higher intakes, especially when prepared with high-fluoride water [19–21]. Children and infants retain higher proportions of absorbed fluoride compared to adults, i.e., about 80–90%, as compared to about 50–60% in adults [6, 15].

As drinking water is usually the major source of exposure, the community water-fluoride concentration has often been used as an exposure parameter in ecological studies. For individual exposure assessment, the total fluoride intake can be calculated from daily water consumption and the intakes of other major sources, such as tea. Analyses of biological samples, i.e., urine and blood (generally in the form of plasma or serum) provide information on fluoride circulating in the body [22]. In adults, the fasting plasma-fluoride concentration, when expressed in micromoles per liter [μmol/L], is approximately equal to the concentration in the drinking water or in the urine expressed in mg/L [1]. Fluoride excretion is mainly via urine, and the concentration represents both recent absorption and releases from long-term accumulation due to continuous bone tissue remodeling [6]. Pregnant women may show lower urinary fluoride levels than non-pregnant controls, perhaps due to fetal uptake and storage in hard tissues [23], although the urinary fluoride excretion tends to increase from the first to the third trimester [11, 24]. Children have lower urine-fluoride concentrations, most likely due to fluoride incorporation in the growing skeleton [1].

As indicator of daily intake [25, 26], urinary fluoride excretion is often assessed in spot urine samples, although morning urine or 24-h samples may provide better precision, as may be the case with timed excretion [27]. To adjust for temporal differences in urine production, fluoride concentrations in spot samples are usually standardized according to the creatinine concentration and/or relative density. These considerations are important when evaluating the validity of exposure assessments in epidemiological studies.

While the blood-brain barrier may to some extent protect the adult brain from many toxic agents, this protection is less likely in the fetus and small child with an incompletely formed barrier [28]. As indication that fluoride passes the blood-brain barrier, fluoride concentrations in human cerebrospinal fluid approach those occurring in serum [29]. Further, imaging studies of radioactive fluoride used in cancer treatment document that circulating fluoride reaches the brain [30–33]. Within the brain, fluoride appears to accumulate in regions responsible for memory and learning [34, 35].

As fluoride can pass both the placental barrier and the blood-brain barrier, it reaches the fetal brain [36]. Accordingly, autopsy studies in endemic areas in China have shown elevated fluoride concentrations in aborted fetal tissues, including brain [37, 38]. Also, fluoride concentrations in maternal and cord serum correlate well [39], cord blood showing slightly lower concentrations, apparently about 80% of the concentrations in maternal serum [40], though depending on gestational age [17]. Fetal blood sampling techniques have allowed documentation of elevated fluoride concentrations in the fetal circulation after administration of sodium fluoride to the mother [41]. Accordingly, assessment of fluoride in maternal samples during pregnancy may be used as indicator of fetal exposure.

Due to a well-established dose-response relationship between early-life fluoride exposure and the degree of dental fluorosis [6, 20, 42], this abnormality can serve as a useful biomarker of developmental fluoride exposure. When water fluoridation was first introduced in the middle of the twentieth century, U.S. health authorities estimated that less than 10% of children in fluoridated communities (at 1 mg/L water) would develop dental fluorosis, and only in its mildest forms [43]. Subsequent epidemiological studies have demonstrated prevalence and severity of fluorosis much higher than predicted [9, 44, 45]. Increased occurrence of dental fluorosis has also been recorded in fluoridated areas in the United Kingdom [46]. This increase may be related to the widened use of fluoridated water for beverages and food products for general consumption and for formula preparation for infants [19, 21], as well as increased usage (and ingestion) of fluoride-containing toothpastes among preschoolers [47].

### Experimental neurotoxicity

In vitro studies have documented fluoride toxicity to brain cells, most of the studies using high fluoride concentrations, though some effects have been demonstrated at lower, more realistic levels [48, 49]. In the low-dose studies, 0.5 μmol/L (10 μg/L) was sufficient to induce lipid peroxidation and result in biochemical changes in brain cells [48], while 3 μmol/L (57 μg/L) induced inflammatory reactions in brain cells [49]. These concentrations are similar to the upper ranges of serum-fluoride levels reported in the general population [6]. In addition, fluoride can negatively affect brain development in rats at levels below those that cause dental lesions [50].

Utilizing computerized surveillance of rat behavior, a landmark study showed signs of neurotoxicity at elevated fluoride exposure [51], and more recent studies have reported fluoride-induced neurochemical, biochemical, and anatomic changes in the brains of treated animals, although often at doses much above human exposure levels. Among possible mechanisms of developmental neurotoxicity is toxicity to the thyroid gland [52], a mechanism relevant in regard to several neurotoxicants [53, 54]. Thus, the NRC concluded that fluoride is an endocrine disrupter that can affect thyroid function at intake levels as low as 0.01 to 0.03 mg/kg/day in individuals with iodine deficiency [1].

A 2016 review by the National Toxicology Program (NTP) focused on fluoride neurotoxicity in regard to learning and memory [55]. At water concentrations higher than 0.7 mg/L, NTP found a low-to-moderate level of evidence. The evidence was the strongest (moderate) in animals exposed as adults and weaker (low) in animals exposed during development, where fewer studies were available at relevant exposure levels. Most experimental studies had used concentrations exceeding the levels added to water in fluoridation programs, but the NTP recognized that rats require about five times more fluoride in their water to achieve the same serum-fluoride concentrations as humans [55].

Subsequently, several additional developmental studies have been published, including two that reported impaired learning/memory in rats consuming water with fairly low fluoride concentrations [56, 57]. However, not all studies have reported adverse effects [58], perhaps due in part to strain or species-related differences in vulnerability to fluoride. In addition, most animal studies used subchronic exposure scenarios and, due to the lack of fluoride transfer into milk, neonatal exposure was not considered, thereby likely underestimating the effect from early-life exposure. Overall, the experimental evidence of developmental neurotoxicity appears to be strengthened and to provide plausibility to the potential occurrence of neurodevelopmental effects in humans.

## Methods

Publications on fluoride neurotoxicity in humans were identified from the PubMed data base by using “fluoride” along with search terms “neurotoxic*”, “neurologic”, and “intelligence”. The searches were narrowed by limiting to “human,” “most recent 10 Years,” and “English.” Additional searches using “fluoride” also included search terms “prenatal exposure delayed effects”[MeSH] or “neurotoxicity syndrome”[MeSH]. Secondary searches used combinations of fluoride with “maternal exposure” or “academic disorder, developmental”.

Supporting literature from earlier years was obtained by using the terms “occupational exposure” or “endemic disease”. References cited in the publications and in recent review reports [55, 59–61] were also retrieved, as were publications listed by PubMed under “Similar articles”. Because these articles may not represent an exhaustive list of relevant studies, separate searches included the web site of the journal *Fluoride* (http://www.fluorideresearch.org/) and the site (http://oversea.cnki.net/kns55/default.aspx) that covers many Chinese-language journals not included in PubMed. Full-text copies of all relevant studies were obtained, and studies were disregarded if no more than an abstract in English was available.

For the purpose of identifying safe exposure levels, regulatory agencies routinely use benchmark dose calculations [62]. While such calculations would normally require access to the original data, approximate BMD and BMDL results can be generated from descriptive data on associations between maternal urinary fluoride concentrations and the child’s IQ [63]. The benchmark dose (BMD) is the dose leading to a pre-defined change (denoted BMR) in the response (in this case, an IQ loss), when compared to comparable, but unexposed individuals. The BMR must be defined before the analysis [62], and recent practice suggests that a decrease in IQ of one point is an appropriate BMR [64–67].

In the above framework, the difference between the expected IQ level at the unexposed background (E [Y (0)]) and at the BMD (E [Y (BMD)]) is equal to the BMR:

$$ \mathrm{E}\ \left[\mathrm{Y}\ (0)\right]-\mathrm{E}\ \left[\mathrm{Y}\ \left(\mathrm{BMD}\right)\right]=\mathrm{BMR} $$

In a linear model (Y(d) = α + βd + ɛ), we get BMD = −BMR/β. The main result of the benchmark analysis is the benchmark dose level (BMDL), which is defined as a lower one-sided 95% confidence limit of the BMD. In the linear model

$$ \mathrm{BMDL}=-\mathrm{BMR}/{\upbeta}_{\mathrm{lower}} $$

where β_lower_ is the one-sided lower 95% confidence limit for β [67]. Thus, in this model the benchmark results are a function of statistics routinely calculated in regression analysis.

For a linear dose-response model, epidemiological studies that report developmental fluoride exposure in regard to IQ will allow computation of BMD and BMDL based only on the regression coefficient and its uncertainty, assuming a Gaussian distribution.

## Results

### Occupational and endemic area studies

The neurotoxicity of chemicals is often first discovered from workplace exposures [68], later followed by case reports and small studies of highly-exposed children or pregnant women, then confirmed in population studies that are later complemented by prospective studies [69]. The same seems to be true of fluoride. A brief summary is therefore presented on the progress of this evidence before focusing on developmental exposures.

In connection with his seminal studies of occupational fluoride poisoning in the 1930s, Kaj Roholm reported evidence of nervous system effects in the Copenhagen cryolite workers [70]: “The marked frequency of nervous disorders after employment has ceased might indicate that cryolite has a particularly harmful effect on the central nervous system.” (p. 178). Later on, the Manhattan Project in the 1940s recorded neurological effects in workers exposed to uranium hexafluoride gas (UF_6_), and the “rather marked central nervous system effect with mental confusion, drowsiness and lassitude as the conspicuous features” was attributed to the fluoride rather than uranium [71].

Subsequent occupational health studies are somewhat harder to interpret, as fluoride exposure usually occurs as part of a mixture, e.g., in aluminum production [72]. Nonetheless, industrial fluorosis (a.k.a. osteosclerosis) was found to be associated with gradually progressive effects on the normal function and metabolism of the brain and other aspects of the nervous system [73], and a review highlighted difficulties with concentration and memory accompanied by general malaise and fatigue [74]. More recent studies have applied neuropsychological tests to assess cognitive problems associated with occupational fluoride exposures [75, 76]. The present literature search did not reveal any recent publications on neurotoxicity from occupational fluoride exposure. While Roholm [70] described unusually serious dental fluorosis in a son of a female cryolite worker, none of the occupational studies identified referred to adverse neurobehavioral effects in the progeny of female workers.

Opportunities for epidemiological studies of the general population depend on the existence of comparable groups exposed to different and stable amounts of fluoride, e.g., from drinking water. Such circumstances are difficult to find in many industrialized countries, as water-fluoride concentrations may not be well defined, residents may consume beverages from a variety of sources, and exposures are affected by residences changing over time. Multiple epidemiological studies of developmental fluoride neurotoxicity have been conducted in countries such as China where elevated water-fluoride concentrations may exceed 1 mg/L in many rural communities. In these settings, families typically remain at the same residence, with a well-defined water source that has provided fairly constant fluoride exposures.

Studies from high-fluoride endemic areas in China have reported on abnormal neuropathology findings from aborted fetuses [37] and lower nerve cell numbers and volumes in fetal brain tissue at the elevated exposures [38]. Deviations observed in neurotransmitters and receptors have suggested neural dysplasia [77], as later replicated along with decreased excitatory aspartic acid and elevated inhibitory taurine in comparison to controls [78]. Although these studies are in agreement with the notion that fluoride from the mother’s circulation can pass into the fetal brain with subsequent anatomic and biochemical changes, the studies related to elevated fluoride exposure originate primarily from coal burning, which may have contributed other, undocumented contaminants.

Additional community studies in adults have focused on cognitive problems and neurological symptoms in subjects with skeletal fluorosis. Using neuropsychological tests, including the Wechsler scale, 49 adult fluorosis patients were compared with controls and showed deficits in language fluency, recognition, similarities, associative learning, and working memory [79]. Further, cognitive impairment in elderly subjects from a waterborne fluorosis area was found to be much more common than in less-exposed controls [80]. Dementia diagnosis in North Carolina was more common at higher water-fluoride concentrations [81], and similar findings for fluoride (and aluminum) have recently been reported from Scotland [82]. Excess occurrence of neurological symptoms (i.e., headaches, insomnia, and lethargy) have also been recorded in both adults and children from waterborne fluorosis areas [83]. However, these studies are hard to evaluate due to uncertainty about past fluoride exposure levels and the possible influence of other risk factors. The literature search did not reveal any other recent studies that added important evidence in this regard.

### Cross-sectional studies of children in exposed communities

Most studies that have investigated fluoride’s impact on childhood IQ are from locations in China with elevated exposure to fluoride, within and outside of known endemic areas [1, 4, 84]. When water supplies derive from springs or mountain sources, small or large pockets of increased exposures may be created near or within similar areas of lower exposures, thus representing useful epidemiology settings. The fluoride exposure from the household water would then represent the only or major difference between nearby neighborhoods. At the time, children in rural China had very little exposure to fluoridated dental products [85]. The local water-fluoride concentration can then serve as a feasible and appropriate exposure parameter, and some studies emphasized that the children were born in the particular study area, and/or had been using the same water supply since birth. Reliable exposure assessment then becomes possible when rural families remain for a long time at the same residence. Any deviation from stable exposure would result in exposure misclassification and thereby a likely underestimation of the toxicity [86]. Thus, the consistency of study findings supports the likelihood that developmental fluoride exposure causes cognitive deficits [4]. Although the study designs are technically cross-sectional, many of the settings allowed consideration of the current exposure as an indicator also of a longer-term exposure level.

Most study reports have not been widely disseminated and considered in literature reviews. Four studies from China that were published in English [87–90] were cited in the 2006 NRC report [1], while the World Health Organization (WHO) considered only two [87, 90] in its revised Environmental Health Criteria document on fluoride from 2002 [26]. A meta-analysis from 2007 included five studies [91], four of which were not in a subsequent review [84]. The latter review was cited by the EU Scientific Committee on Health and Environmental Risks (SCHER) working group in 2010 [5] in support of a conclusion that the evidence of neurotoxicity was insufficient.

A meta-analysis from 2012 was based on a collaboration with Chinese experts on fluoride toxicity and covered 27 cross-sectional studies reporting associations between children’s intelligence and their fluoride exposure [4]. Overall, children who lived in areas with high fluoride exposure had lower IQ scores than those who lived in low exposure or control areas, the average difference being close to 7 IQ points. These findings were consistent with an earlier review [84], but included nine more studies and more systematically addressed study selection, exclusion information, and bias assessment.

Two of the 27 studies that we included in the analysis were conducted in Iran [92, 93], while all other study populations were from China. Two cohorts were exposed to fluoride from coal burning [94, 95], but otherwise the study populations were exposed to fluoride through drinking water contaminated from soil minerals. Due to the use of different cognitive tests, normalized data were used to estimate the possible effects of fluoride exposure on intelligence. The results were materially unchanged in various sensitivity analyses, as were analyses that excluded studies with possible concerns about co-factors, such as iodine deficiency and arsenic toxicity, or non-water fluoride exposure from coal burning [4].

Among the 27 studies, all but one showed random-effect standardized mean difference (SMD) estimates that indicated an inverse association, ranging from − 0.95 to − 0.10 (one study showed a slight, non-significant effect in the opposite direction). The overall random-effects SMD estimate (and 95% confidence interval, CI) was − 0.45 (− 0.56, − 0.34). Given that the standard deviation (SD) for the IQ scale is 15, an SMD of − 0.45 corresponds to a loss of 6.75 IQ points. Although substantial heterogeneity was present among the studies, there was no clear evidence of publication bias [4]. Given the large number of studies showing cognitive deficits associated with elevated fluoride exposure under different settings, the general tendency of fluoride-associated neurotoxicity in children (*p* < 0.001) seems robust.

### Recent cross-sectional studies of children

The present study presents an updated literature search that revealed 14 new studies on the association between early-life fluoride exposure and IQ in children (Table 1). All 14 studies reported apparent associations between elevated fluoride exposure and reduced intelligence, although one did not reach statistical significance. The several new Chinese-language studies showed similar associations between fluoride exposure and reduced IQ [96, 101–103, 105, 107, 108], although often published as short reports in national journals and according to the standards of science at the time. Similar findings were reported from India [98, 100, 110] and Africa [104, 106]. As with the previous reports, most of these newer studies suffer from limitations of covariate reporting, which limited the opportunity to assess possible bias. Also, a variety of outcomes have been employed, such as neuropsychological tests and Raven-based intelligence scales. Of note, fluoride exposure was accompanied by other contaminants from coal burning in some studies [96, 99, 101, 102]. Four studies used the degree of dental fluorosis as exposure parameter, and three of them reported a clear negative association with IQ [100, 103, 107], although statistical significance was not reached in one study [102]. The water-fluoride concentrations tended to be somewhat lower than in previous studies and thus more relevant to exposures occurring outside of endemic areas.

**Table 1:** Characteristics of 14 cross-sectional studies of fluoride exposure and children’s cognitive and developmental outcomes published after 2012

Reference	Study location, year	No. in high-exposure group	No. in reference group	Age range (or mean), years	Fluoride exposure		Outcome measure	Results
					Assessment	Mean or range (mg/L)		
[96]	China, 2014	123	42	8–12	Urine	3.03 (urine, short-term); 2.33 (urine, long-term); 1.34 (urine, ref)	RSPM^a^	Fluoride exposure was negatively associated with children’s IQ
[97]	China, 2015	26 (moderate/severe dental fluorosis)	8 (normal/questionable dental fluorosis)	6–8	Drinking water, urine	2.66 (water, moderate/severe); 1.0 (water, normal/questionable); 2.44 (urine, moderate/severe); 0.45 (urine, normal/questionable)	WRAML^b^; WISC-IV^c^	Moderate and severe fluorosis were significantly associated with deficits in digit span scores.
[98]	India, 2015	215	214	6–12	Drinking water	2.41 (water, high); 0.19 (water, ref)	RCPM^d^	IQs of highly exposed children were significantly lower than those with low-level exposure
[99]	China, 2015	84	96	7–13	Drinking water, urine	1.40 (water, high); 0.63 (water, ref); 2.40 (urine, high); 1.10 (urine, ref)	CRT-RC^e^	Fluoride exposure was negatively associated with children’s IQ
[100]	India, 2016	23 (severe dental fluorosis)	4 (normal dental fluorosis)	6–18	Groundwater and urine	2.11 (water); 0.45–17.00 (range, urine)	CRT-RC^e^	IQ was negatively correlated with degree of dental fluorosis
[101]	China, 2017	68	50	3–12 months	Coal burning vs. control	Mothers in exposed group had dental fluorosis	MDI & PDI (CDCC)^f^	MDI & PDI in exposed group were significantly lower than those in the control group
[102]	China, 2017	167	120	8–12	Coal burning vs. control	Dental fluorosis index 53.9% in exposed group	RSPM^a^	IQ was lower in children with high fluoride exposure (not significant)
[103]	China, 2018	221	100	8–12	Drinking water	1.2 (water, high); 0.25 and 0.78 (water, controls)	CRT-RC^e^	IQ was lower in children from endemic areas and in those with dental fluorosis
[104]	Sudan, 2018	775 (total)	N/a	6–14	Drinking water	0.01–2.07 (water)	School performance based on method adopted by MoE	Inverse relationship between fluoride in drinking water and average school performance
[105]	China, 2018	134	134	8–12	Dental fluorosis	N/a	CRT-RC^e^	IQ was lower in children from endemic areas
[106]	Egypt, 2018	186	814	4.6–11	Drinking water	0.92–3.75 (water)	DAP^g^	Decreased scores in children from areas with elevated fluoride in drinking water
[107]	China, 2018	1250	1636	7–13	Drinking water and urine	2.00 + 0.75 (water, high); 1.37 + 1.08 (urine, high); 0.50 + 0.27 (water, ref); 0.41 + 0.49 (urine, ref)	CRT-RC2^e^	IQ was lower in children at higher fluoride in water and urine and at greater severity of dental fluorosis
[108]	China, 2019	25	27	8–12	Drinking water	N/a	CRT-RC2^e^	IQ was lower at elevated fluoride exposure
[109]	China, 2020	571 (total)	N/a	7–13	Drinking water, urine	1.39 + 1.01 (water); 1.28 + 1.30 (urine)	CRT-RC2^e^	Low to moderate fluoride exposure is associated with alterations in thyroid function and lower IQ

^a^Raven’s Standardized Progressive Matrices; ^b^Wide Range Assessment of Memory and Learning; ^c^Wechsler Intelligence Scale for Children-Revised; ^d^Raven’s Colored Progressive Matrices; ^e^Combined Raven’s Test-The Rural in China; ^f^mental development index & psychomotor development index (assessed using the Standardized Scale for the Intelligence of Children formulated by the Children Development Center of China; ^g^DAP, Draw-A-Person

To ascertain the validity of the methodology used in Chinese studies of fluoride neurotoxicity, my colleagues and I carried out a small study in Sichuan using methods commonly applied in Western neurobehavioral epidemiology [97]. The 51 children examined had lived in their respective communities all their life, i.e., at least since conception. All three measures of fluoride exposure showed negative associations for cognitive function tests. One exposure parameter was the known water-fluoride concentration at the residence where the child was born, another was the child’s morning urine-fluoride after having ingested fluoride-free water the night before (neither measure reached formal statistical significance as predictor of cognitive deficits). The strongest and statistically significant association was seen with the degree of dental fluorosis that served as a marker of the child’s early-life fluoride exposure. Other recent studies (Table 1) also found dental fluorosis to be a useful risk indicator. While one previous study in the U.S. failed to observe a relationship between dental fluorosis and behavior (parental assessment by the Child Behavior Checklist) [111], a dose-response relationship between urinary fluoride concentrations (range, 0.24–2.84 mg/L) and reduced IQ was reported in a population without any severe dental fluorosis [112].

A recent meta-analysis of waterborne fluoride exposures [60] covered 18 studies with water-fluoride concentrations below 4 mg/L; clear IQ reductions were observed at water-fluoride concentrations of about 1 mg/L and above. In addition, four cross-sectional studies reported linear relationships between urinary fluoride (one study also included plasma-fluoride) and IQ among children living in areas with mean water-fluoride contents of 1.4 mg/L, 1.5–2.5 mg/L, 1.4 mg/L, and 0.5–2.0 mg/L [99, 107, 109, 113].

Although meta-analysis of studies has previously been carried out [4, 60], the heterogeneity of the new studies and differences in exposure assessment and cognitive tests suggested that a joint analysis would require too many assumptions to provide useful evidence on the dose-dependence of neurotoxicity. The information summarized in Table 1 therefore serves as qualitative documentation that elevated fluoride exposure during early development is associated with cognitive deficits. Although the presence of confounding bias cannot be excluded, the fairly uniform findings under different study conditions would argue against any serious bias. The largest study, by far, reported an IQ loss of 4.29 (95% CI, 0.48–8.09) and 2.67 (0.68–4.67) for each increase by 0.5 mg/L in the fluoride concentration in water and urine, respectively [107]. A recent study with individual exposure data [109] reported lower losses of 0.79 (0.28–1.30) and 0.61 (0.22–0.99) IQ points for each increase by 0.5 mg/L in fluoride in water and urine, respectively. Of note, the ranges of exposures in these studies overlap with concentrations commonly reported from regions without endemic disease.

### Prospective studies

More weight must be placed on prospective studies that include assessment of individual levels of fluoride exposures in early life (Table 2). Two prospective studies from New Zealand explored the possible neurobehavioral consequences of community water fluoridation. The first study reported no association between behavioral problems and residence in a fluoridated community during the first 7 years of life [114]. However, like the subsequent study, the authors had no access to individual measurements of fluoride exposure, and the exposure status relied solely on residence in a fluoridated community and its duration, where age at the time of residence was apparently not considered.

**Table 2:** Characteristics of the five prospective studies of fluoride exposure and children’s cognitive and neurobehavioral, developmental and cognitive outcomes

Reference	Study location, year	No. in high-exposure group	No. in reference group	Age range (or mean), years	Fluoride exposure		Outcome measure	Results
					Assessment	Range or mean (mg/L)		
[114]	New Zealand, 1986	1028 (total)	N/a	0–7	Drinking water fluoridation	N/a	RBS^a^ and CBRS^b^	No association between duration of residence in fluoridated community and behavioral problems
[115]	New Zealand, 2015	992 (total)	N/a	5 and 7–13	Water fluoridation, supplements	N/a	WISC^c^	No significant association found between tablet use, use of fluoride toothpaste, or childhood community water fluoridation and IQ, respectively
[63]	Mexico, 2017	287 (total)	N/a	4 and 6–12	Maternal urinary fluoride (MUF)	0.88 (mean)	MSCA^d^; WASI^e^	Higher MUF levels were associated with lower scores on cognitive function tests in offspring
[24]	Mexico, 2017	211 (total)	N/a	3–15 months	Drinking water and MUF	0.5–12.5 (water); 0.16–4.9 (MUF, 1st trimester); 0.7–6.0 (MUF, 2nd trimester); 1.3–8.2 (MUF, 3rd trimester)	BSDI-II^f^	MUF levels sampled during the 1st and 2nd trimesters were inversely associated with mental development in infants
[116]	Canada, 2018	275 (city fluoridation)	335 (no city fluoridation)	3.4	MUF, fluoride intake	0.06–2.44	WPPSI-III^g^	Higher MUF levels predicted lower IQ in males but not females; higher maternal fluoride intake predicted lower IQ

^a^Rutter Behavior Rating Scales; ^b^Connors Behavior Rating Scales; ^c^Wechsler Abbreviated Scale of Intelligence; ^d^McCarthy Scales of Children’s Abilities; ^e^Wechsler Abbreviated Scale of Intelligence; ^f^Bayley Scale of Infant Development II; ^g^Wechsler Preschool and Primary Scale of Intelligence, 3rd edition

A more comprehensive study was based on a birth cohort established in Dunedin, New Zealand from births in 1972–1973 [115]. The 1037 children were recruited at age 3 years, and IQ tests were administered at ages 7, 9, 11 and 13 years, and again at age 38; the average IQ result for 992 subjects was used for comparison between residents in areas with and without water fluoridation. No significant differences in IQ in regard to fluoridation status were noted, and this finding was independent of potential confounding variables that included sex, socioeconomic status, breastfeeding, and birth weight. Prenatal fluoride exposure was not considered. The average difference in childhood exposure between fluoridated vs. non-fluoridated areas was estimated to be 0.3 mg/day [117]. However, the 93 cohort subjects who did not live in a fluoridated area may well have received fluoride supplements, as was the case for a total of 139 children in the study, thereby impacting on the exposures [20]. A further concern is that formula may have contributed substantial fluoride exposure [19, 21], and it is therefore interesting that breastfeeding – and thus avoidance of formula – in the fluoridated areas contributed an advantage that averaged 6.2 IQ points at age 7–13 years, while the advantage was less (4.3) in the non-fluoridated areas [115]. Subsequently, the authors estimated the average total fluoride intake up to age 5 years, including tablets, toothpastes, and dietary sources, without finding any IQ difference [118]. However, information on maternal tea consumption during pregnancy was not obtained, although tea has long been recognized as an important source of fluoride in New Zealand [119]. Lead exposure in this cohort was later reported to cause IQ deficits [120], but control for the blood-lead concentration at age 9 years showed no change in the results for fluoride [117]. Despite the shortcomings, this study has been hailed as evidence that fluoridated water is “not neurotoxic for either children or adults, and does not have a negative effect on IQ” [121]. This conclusion seems rather optimistic [122], given the fact that the exposure assessment was imprecise (especially for prenatal exposure) and that the statistical power was probably insufficient to allow identification of any important IQ deficit.

More recent studies provide more robust evidence. In a prospective study from an area in Mexico with elevated levels of fluoride in drinking water, maternal pregnancy urine-fluoride (corrected for specific gravity) was examined for its association with scores on the Bayley Scales among 65 children evaluated at age 3–15 months [24]. The mothers in the study had average urine-fluoride concentrations at each of the three trimesters of pregnancy of 1.9, 2.0, and 2.7 mg/L (higher than the following study). The fluoride exposure indicators during first and second trimesters were associated with significantly lower scores on the Bayley Mental Development Index score after adjustment for covariates [24].

The existence of the ELEMENT (Early Life Exposure in Mexico to Environmental Toxicants) birth cohort allowed longitudinal measurements of urine-fluoride in pregnant mothers and their offspring and their associations with measures of cognitive performance of the children at ages 4 and 6–12 years [63]. The cohort had been followed to assess developmental lead neurotoxicity, and biobanked urine samples were available for fluoride analysis and adjustment for creatinine and density. Most of the mothers provided only one or two urine samples, thereby introducing some imprecision in the exposure estimate. Child cognitive function was determined by the General Cognitive Index (GCI) of the McCarthy Scale at age 4 years in 287 children, and IQ by an abbreviated Wechsler scale (WASI) at age 6–12 years in 211 children. Urinary fluoride (mg/L) in the mothers averaged 0.90 (s.d., 0.35) and, in the children, 0.82 (s.d., 0.38). Covariates included gestational age, birth weight, sex, parity, age at examination, and maternal characteristics, such as smoking history, marital status, age at delivery, maternal IQ, and education. After covariate adjustment, an increase in maternal urine-fluoride by 1 mg/L during pregnancy was associated with a statistically significant loss of 6.3 (95% CI, − 10.8; − 1.7) and 5.0 (95% CI, − 8.2; − 1.2) points on the GCI and IQ scores, respectively. These associations remained significant, and the effect sizes appeared to increase, in sensitivity analyses that controlled for lead, mercury, and socioeconomic status.

Although adjustment could not be made for iodine deficiency or arsenic exposure, any residual confounding was judged to be small in this population. Important strengths are that the cohort was followed from birth with meticulous documentation for lead exposure and other neurobehavioral risks. This study also ascertained the childhood fluoride exposure at the time of IQ testing (6–12 years) and found no indication of adverse impact on the IQ in the cross-sectional analysis [63].

Between 2008 and 2011, 2001 pregnant women were recruited into the Maternal-Infant Research on Environmental Chemicals (MIREC) cohort in Canada. A subset of 601 of their children were examined at age 3–4 years, slightly less than half of them residing in fluoridated communities [116]. Maternal spot urine samples were obtained from each of the three semesters of pregnancy, and results were analyzed for those 512 mother-child pairs where urine was available from all three semesters, so that the overall average urine-fluoride could be used as an exposure biomarker, with adjustment for specific gravity and creatinine. Information was obtained on food and beverage intakes, including tea (assuming a fluoride content of 0.52 mg in each cup of black tea). Intellectual abilities were assessed using the age-appropriate Wechsler scale that provided a full-scale IQ. Covariate adjustment included exposures to other neurotoxicants and other relevant covariates, such as sex, age at examination, and maternal exposure to indirect smoking, race, and education [116]. As had been shown by the same research group in a previous study of a larger population [11], women residing in fluoridated communities had higher urine-fluoride concentrations (0.69 vs 0.40 mg/L) and also higher calculated daily fluoride intakes from water and other beverages (0.93 vs. 0.30 mg/day). Regression analyses showed that an increase in *urine*-fluoride of 1 mg/L was associated with a statistically significant loss in IQ of 4.49 points in boys, though not in girls. An increase of 1 mg/L of fluoride in *water* and an increase of 1 mg/day of fluoride *intake* was associated with an IQ loss of 5.3 points and 3.66 points, respectively, for both boys and girls [116]. Thus, this study at somewhat lower exposures is in good agreement with the data from the two studies carried out in Mexico.

In an extension of the MIREC study of prenatal fluoride exposures, the authors subsequently assessed the possible impact of fluoride exposure from reconstituted formula in fluoridated and non-fluoridated communities [123]. After adjustment for prenatal fluoride exposure and other covariates, each increase by 1 mg/L in the water fluoride concentration was found to be associated with a statistically significant decrease of 8.8 IQ points in the children who had been formula-fed in the first 6 months of life, while no such difference was seen among the exclusively breastfed children. Although the results were somewhat unstable and included only 68 formula-fed children from fluoridated communities, these results support the notion that early postnatal brain development is also likely to be vulnerable to neurotoxicant exposures, as is well documented, e.g., from arsenic exposure in infancy [124].

The substantial IQ losses associated with elevated water-fluoride concentrations are in accordance with the difference of almost 7 IQ points between exposed groups and controls in the meta-analysis from 2012 [4]. Also, the largest cross-sectional study from 2018 showed a statistically significant loss of 8.6 IQ points for each increase by 1 mg/L in the fluoride concentration in water [107], although somewhat less in another recent study [109].

Several additional reports using other cognition measures are also of relevance. Another Canadian study analyzed data from two cycles of the Canadian Health Measures Survey (CHMS) [125]. Randomly measured urine-fluoride results from children aged 3-to-12 years were linked to parental reports or self-reported learning disabilities. When the two cycles of the CHMS were combined (both including at least 1100 subjects), unadjusted urine-fluoride was significantly correlated with an increased incidence of learning disabilities. However, this effect lost its statistical significance after controlling for creatinine and specific gravity. The authors concluded that there was no robust association between fluoride exposure and reported learning disability among Canadian children at the ages studied. However, the exposure assessment probably did not reflect the time of greatest vulnerability to fluoride, and the information on learning disability was somewhat uncertain, also in regard to the time of appearance. A more recent analysis relying on the same data showed that elevated fluoride in tap water was associated with an increased risk of Attention-Deficit/Hyperactivity Disorder (ADHD) symptoms and ADHD diagnosis among Canadian youth, although the association with ADHD was not present when urine-fluoride concentrations were used as exposure indicator [126].

A related study of the ELEMENT population showed that elevated prenatal fluoride exposure was associated with higher scores on the Conners’ Rating Scale and thus with tendencies toward inattention and development of ADHD [127].

These prospective studies from North America focused on prenatal and early postnatal exposure known as a key window of neurological vulnerability [69]. All of these studies relied on individual exposure indicators, thus providing substantial support to the conclusion that elevated fluoride exposure during early development can cause neurotoxicity.

### Retrospective studies of fluoride neurotoxicity

A few retrospective studies are available but provide only weak evidence on the possible existence of fluoride-related neurotoxicity. A Swedish study utilized the register of military conscripts who underwent neurocognitive tests [128]. The authors then estimated the water-fluoride concentrations for each of the about 80,000 subjects based on their residential history, where the geographic location of the current residence was linked to a water supply with a known fluoride content. The study found no meaningful or consistent relationship between the test results and the home water-fluoride concentration (0 to 2 mg/L). The study did identify a relationship between water-fluoride and increased income, which the authors attributed to improved dental health. However, the study did not have access to specific individual fluoride exposure data, nor was developmental exposure assessed. This study is therefore non-informative due to the likely misclassification of any causative exposure.

In the U.S., parental reports on ADHD among 4-to-17-year-olds were collected from the National Survey of Children’s Health and combined with information on water fluoridation at state level [129]. The prevalence of artificial water fluoridation in 1992 predicted significantly the state prevalence of ADHD from the surveys in 2003, 2007 and 2011. After adjustment for socioeconomic status, each 1% increase in artificial fluoridation prevalence in 1992 was associated with approximately 67,000 to 131,000 additional ADHD diagnoses from 2003 to 2011. Given the state-level exposure assessment and the use of parental reports of ADHD, this ecological study has important weaknesses, although the findings are in agreement with other recent studies. However, the study has been criticized, as inclusion of mean elevation as a covariate apparently abolishes the significance of fluoridation as a predictor [130].

Overall, the retrospective studies are limited by exposure data that do not necessarily reflect early-life conditions and therefore add little weight to the information otherwise available on fluoride neurotoxicity in children.

### Dose-dependence and benchmark doses

The studies reviewed show dose-dependent fluoride neurotoxicity that appears to be statistically significant at water concentrations of or below 1 mg/L, but the studies themselves do not identify a likely threshold. Regulatory agencies often use benchmark dose calculations to develop non-cancer health-based limits for dietary intakes, such as drinking water [62, 131]. One recent report [132] used this approach to generate benchmark results from a study of more than 500 children in China [89]. The authors used a high BMR of 5 IQ points, but results were also given for a more appropriate BMR of 1 IQ point. For the latter, the BMDL was calculated to be a daily intake level of 0.27 mg/day [132]. Using the average water intake of 1.24 L/day in non-pregnant women [133], the BMDL corresponds to a water concentration of 0.22 mg/L. The report did not provide data for urine-fluoride concentrations.

As described in the Methods section, the regression coefficients and their standard deviations, as provided in the published reports [63, 116], were applied to estimate tentative BMD values. Assuming linearity and Gaussian distributions, the right-hand columns of Table 3 show the calculated results for the two prospective studies with the maternal urine-fluoride concentration as the exposure parameter in regard to the cognitive function measures (both boys and girls). For the ELEMENT study, results for the larger number of children with CGI outcomes are also shown. Overall, the BMDL results appear to be in agreement.

**Table 3:** Adjusted differences in cognitive outcomes per mg fluoride per liter maternal urine (U-fluoride) during pregnancy, and benchmark dose results (boys and girls) in regard to maternal urinary fluoride excretion (mg/L urine adjusted for creatinine)

Study	Reference	Number	Outcome	U-fluoride (median)	Estimate	95% CI	BMD	BMDL
ELEMENT	[63]	287	GCI	0.84	−6.3	−10.8; − 1.7	0.16	0.10
ELEMENT	[63]	211	IQ	0.82	−5.0	−8.2; − 1.2	0.20	0.13
MIREC	[116]	512	IQ	0.51	−2.0	− 5.2; 1.3	0.51	0.21

The Table 3 results also appear to be reliable, given that the studies provide ample coverage of subjects with lower-level exposures close to the BMDL. The Canadian children had lower prenatal exposures than the Mexican study subjects, and along with the apparent lack of fluoride effects in girls, the BMD results are higher than in the ELEMENT study, although the greater uncertainty results in a fairly low BMDL. The results suggest a BMDL of about 0.2 mg/L or below, a level that is similar to the result calculated from the study in China [89, 132] and clearly below commonly occurring exposure levels, even in communities with drinking water fluoridation.

## Plausibility and implications

The present review updates the conclusions from a 2012 meta-analysis of cross-sectional studies of intellectual deficits associated with elevated fluoride exposure [4]. Subsequent epidemiological studies have strengthened the links to deficits in cognitive functions, several of them providing individual exposure levels, though most of the new studies were cross-sectional and focused on populations with fluoride exposures higher than those typically provided by fluoridated water supplies. Prospective studies from the most recent years document that adverse effects on brain development happen at elevated exposure levels that occur widely in North America and elsewhere in the world, in particular in communities supplied with fluoridated drinking water [24, 63, 116, 123]. These new prospective studies are of very high quality and, given the wealth of supporting human studies and biological plausibility, leave little doubt that developmental neurotoxicity is a serious risk associated with elevated fluoride exposure, especially when this occurs during early brain development. While evidence on the neurotoxic impact of early postnatal exposure remains limited [21, 123], other neurotoxicity evidence suggests that adverse effects are highly plausible [124].

Research on laboratory animals confirms that elevated fluoride exposure is toxic to the brain and nerve cells, as already indicated by the NRC review [1]. The evidence today is substantially more robust. The NTP review placed more confidence in fluoride impairing learning in *adult* animals due to fewer experimental studies being available on developmental exposure [55]. Still, not all studies are in agreement [58], perhaps due to species or strain differences in vulnerability. However, fluoride is known to pass the placental barrier and to reach the brain, and the animal studies bear out the importance of the prenatal period for fluoride neurotoxicity. Toxicant exposures in early life can have much more serious consequences than exposures occurring later in life, and the developing brain is known to be particularly vulnerable [69]. Thus, the vulnerability of early brain development supports the notion that fluoride neurotoxicity during early life is a hazard of public health concern [134].

Dental fluorosis has been dismissed as a “cosmetic” effect only [6, 135, 136], but the association of dental changes with intellectual deficits in children [95, 97, 100, 103, 107, 112] suggests that dental fluorosis should no longer be ignored as non-adverse. Dental fluorosis may perhaps serve as a sensitive indicator of prenatal fluoride exposure, and information is needed to determine to which extent the time windows for dental fluorosis development in different tooth types [137] overlap with vulnerable periods for brain development.

Although the adverse outcome pathway is unclear, several epidemiological studies suggest that thyroid dysfunction is a relevant risk at elevated fluoride exposures. Thus, studies in children have reported deficient thyroid functions, including elevated TSH (thyroid stimulating hormone) at elevated fluoride exposure [138–142], and one study linked elevated fluoride exposure to both thyroid dysfunction and IQ deficits [109]. In Canada, elevated urine-fluoride was associated with increased TSH among iodine-deficient adults, though not in the general population, after exclusion of those with known thyroid disease [143]. In England, the diagnosis of hypothyroidism was nearly twice as frequent in medical practices located in a fully fluoridated area, as compared to non-fluoridated areas [144]. These findings are highly relevant to the neurotoxicity concerns, as thyroid hormones are crucial for optimal brain development [53, 54].

Given that fluoride is excreted only in minute amounts in human milk [1, 18], the focus on prenatal exposure appears justified, but formula-mediated neonatal exposures represent an additional concern, as indicated by dental fluorosis studies [137] and the most recent study from Canada [123]. The human brain continues to develop postnatally, and the period of heightened vulnerability therefore extends over many months through infancy and into early childhood [69]. Fluoride exposures during infancy are of special concern in regard to formula produced with fluoride-containing water [21, 145]. Unfortunately, current animal models do not appropriately cover neonatal fluoride exposure. Thus, future studies that focus on exposures prenatally, during infancy, and in later childhood may allow more detailed assessment of the vulnerable time windows for fluoride neurotoxicity.

One prospective study suggested that boys may be more vulnerable to fluoride neurotoxicity than girls [116]. Given that endocrine disrupting mechanisms often show sex-dependent vulnerability [146], further research is needed to understand the extent that males may require additional protection against fluoride exposure. Recent studies have also identified possible genetic predisposition to fluoride neurotoxicity [113, 147]. This means that some subgroups of the general population will be more vulnerable to fluoride exposure so that exposure limits aimed at protecting the average population may not protect those with susceptible genotypes, as has been shown, e.g., for methylmercury neurotoxicity [148]. The impact of iodine deficiency on fluoride vulnerability also needs to be considered [143].

Past studies of fluoride-exposed workers suggest possible neurotoxicity, but recent evidence rather points to possible accelerated aging in fluoride-exposed adults [80–82]. As has been proposed for other developmental neurotoxicity [134, 149], early-life exposure to fluoride deserves to be examined in regard to its possible impact on the risk of adult neurodegenerative disease.

Despite the growing evidence, health risks from elevated exposures to fluoride have received little attention from regulatory agencies. Thus, the EPA’s regulation of fluoride in water, most recently confirmed in 2016, is based on the assumption that crippling fluorosis is the most sensitive adverse effect [59]. The MCLG for fluoride (4 mg/L) may perhaps serve that purpose, but it is clearly not protective of adverse effects on the brain, especially in regard to early-life exposures. In its most recent review of fluoride [59], the EPA referred to the 2012 meta-analysis [4] and highlighted that IQ deficits occurred at water-fluoride concentrations “up to 11.5 mg/L”, although this level represented only the highest exposure in the 27 studies assessed. Neither the EPA nor a U.S. federal panel [9, 59] noted that most of the studies included in the review had water-fluoride concentrations below the MCLG of 4 mg/L. Thus, out of the 18 studies that provided the water-fluoride concentrations, 13 found deficits at levels below the MCLG, with an average elevated level at 2.3 mg/L, the lowest being 0.8 mg/L [4]. The results in Table 1 show that the recent cross-sectional results from different communities are in accordance with the previous review [4] and extend the documentation of cognitive deficits associated with only slightly elevated exposures.

The appearance of prospective studies that offer strong evidence of prenatal neurotoxicity should inspire a revision of water-fluoride regulations. The benchmark results calculated from these new studies, though tentative only at this point, support the notion that the MCLG is much too high. Depending on the use of uncertainty factors, a protective limit for fluoride in drinking water would likely require that the MCGL be reduced by more than a 10-fold factor, i.e., below the levels currently achieved by fluoridation.

The notion that fluoride is primarily a developmental neurotoxicant means that fluoride – an element like lead, mercury, and arsenic – can adversely affect brain development at exposures much below those that cause toxicity in adults. For lead and methylmercury, adverse effects in children are associated with blood concentrations as low as about 10 nmol/L. Blood-fluoride concentrations associated with elevated intakes from drinking-water may exceed 20 μg/L, or about 1 μmol/L, i.e., about 100-fold greater than the serum concentrations of the other trace elements that cause neurodevelopmental damage. Thus, although fluoride is neurotoxic, it appears to be much less potent than elements that occur at much lower concentrations in the Earth’s crust. Although substances that occur naturally in the biosphere may be thought to be innocuous, or even beneficial as in the case of fluoride, the anthropogenic elevations in human exposures may well exceed the levels that human metabolism can successfully accommodate [150].

Perhaps dentistry interests in promoting water fluoridation have affected the risk assessment and reduced the regulatory attention to fluoride toxicity. Thus, reports on fluoride toxicity have been disregarded under a heading referring to “Anti-Fluoridation Activities” [121], and our review article [4] was said to rely on “selective readings” [115], with IQ deficits occurring at high fluoride concentrations “up to 11.5 mg/L” [151], although most of the studies related to concentrations that were only slightly elevated. Further, an ecological study without individual exposure data [115] that failed to identify an association with IQ was considered as strong support of the safety of water fluoridation and more relevant to fluoridation policy than other evidence on neurotoxicity [121].

While water fluoridation continues to be recommended [9], the benefits appear to be minimal in recent studies of caries incidence [152]. Perhaps due to modern use of topical fluoride products, especially fluoridated toothpaste, countries that do *not* fluoridate the water have seen drops in dental cavity rates similar to those observed in fluoridated countries [153]. This finding is in agreement with the observation that fluoride’s predominant benefit to dental health comes from topical contact with the surface of the enamel, not from ingestion, as was once believed [154, 155]. Already in 2001, the U.S. Centers for Disease Control (CDC) concluded that fluoride supplementation during pregnancy did not benefit the child’s dental health [156]. Consensus has since then been building on the lack of efficacy of water fluoridation in preventing caries [152].

It therefore appears that population-based increase of systemic fluoride exposure may be unnecessary and, according to the evidence considered in this review, counterproductive. The focus should therefore shift from population-wide provision of elevated oral fluoride intake to consideration of the risks and consequences of developmental neurotoxicity associated with elevated fluoride exposure in early life. The prospective studies suggest that prevention efforts to control human fluoride exposures should focus on pregnant women and small children. In addition to drinking water, attention must also be paid to other major sources of fluoride, such as black tea [13]. Thus, excessive tea-drinking is known to potentially cause skeletal fluorosis [12], and the possible impact of tea drinking deserves to be considered along with other possible sources that may affect pregnant women and small children.

The evidence on fluoride neurotoxicity in the general population is fairly recent and unlikely to represent the full toxicological perspective, including adverse effects that may occur at a delay, as has been seen with many developmental neurotoxicants in the past [134]. While some ecological studies failed to identify clear evidence for fluoride neurotoxicity, they cannot be relied on as proof that elevated fluoride exposure is safe, in particular regarding early brain development. Recent prospective studies with individual exposure assessments provide strong evidence, and the large number of cross-sectional studies from populations with stable and well-characterized exposures provide additional support.

## Conclusions

Previous assessment of neurotoxicity risks associated with elevated fluoride intake relied on cross-sectional and ecological epidemiology studies and findings from experimental studies of elevated exposures. The evidence base has greatly expanded in recent years, with 14 cross-sectional studies since 2012, and now also three prospective studies of high quality and documentation of individual exposure levels. Thus, there is little doubt that developmental neurotoxicity is a serious risk associated with elevated fluoride exposure, whether due to community water fluoridation, natural fluoride release from soil minerals, or tea consumption, especially when the exposure occurs during early development. Even the most informative epidemiological studies involve some uncertainties, but imprecision of the exposure assessment most likely results in an underestimation of the risk [86]. Thus, the evidence available today may not quite reflect the true extent of the fluoride toxicity. Given that developmental neurotoxicity is considered to cause permanent adverse effects [69], the next generation’s brain health presents a crucial issue in the risk-benefit assessment for fluoride exposure.

## Data Availability

N/A.

## Abbreviations

ADHD: Attention-Deficit/Hyperactivity Disorder
BMD: Benchmark dose
BMDL: Benchmark dose level
BMR: Benchmark response
BSDI-II: Bayley Scale of Infant Development II
CBRS: Connors Behavior Rating Scales
CHMS: Canadian Health Measures Survey
CI: Confidence interval
CRT-RC: Combined Raven’s Test-The Rural in China
DAP: Draw-A-Person
EFSA: European Food Safety Authority
ELEMENT: Early Life Exposures in Mexico to Environmental Toxicants
EPA: Environmental Protection Agency
GCI: General Cognitive Index
IQ: Intelligence Quotient
MCLG: Maximum Contaminant Level Goal
MeSH: Medical Subject Headings (PubMed)
MIREC: Maternal-Infant Research on Environmental Chemicals
MSCA: McCarthy Scales of Children’s Abilities
NRC: National Research Council
NTP: National Toxicology Program
RBS: Rutter Behavior Rating Scales
RCPM: Raven’s Colored Progressive Matrices
RSPM: Raven’s Standardized Progressive Matrices
SD: Standard Deviation
SMD: Standardized Mean Difference
TSH: Thyroid Stimulating Hormone
WASI: Wechsler Abbreviated Scale of Intelligence
WISC-IV: Wechsler Intelligence Scale for Children-Revised
WPPSI-III: Wechsler Preschool and Primary Scale of Intelligence, 3rd edition
WRAML: Wide Range Assessment of Memory and Learning
